# A trend of osteocalcin in diabetes mellitus research: bibliometric and visualization analysis

**DOI:** 10.3389/fendo.2024.1475214

**Published:** 2025-01-13

**Authors:** Zixu Liu, Yuchen Mao, Kangping Yang, Shukai Wang, Fang Zou

**Affiliations:** ^1^ Department of Endocrinology and Metabolism, The Second Affiliated Hospital of Nanchang University, Nanchang, China; ^2^ The First Clinical Medicine School, Nanchang University, Nanchang, China; ^3^ The Second Clinical Medicine School, Nanchang University, Nanchang, China

**Keywords:** osteocalcin, diabetes mellitus, bibliometric analysis, VOSviewer, visualization

## Abstract

**Background:**

Osteocalcin has attracted attention for its potential role in diabetes management. However, there has been no bibliometric assessment of scientific progress in this field.

**Methods:**

We analysed 1680 articles retrieved from the Web of Science Core Collection (WoSCC) between 1 January 1986 and 10 May 2024 using various online tools.

**Result:**

These papers accumulated 42,714 citations,with an average of 25.43 citations per paper. Publication output increased sharply from 1991 onwards. The United States and China are at the forefront of this research area.

**Discussion:**

The keywords were grouped into four clusters: ‘Differential and functional osteocalcin genes’, ‘Differential expression of osteocalcin genes in relation to diabetes mellitus’, ‘Role of osteocalcin in the assessment of osteoporosis and diabetes mellitus’, and ‘Indirect involvement of osteocalcin in metabolic processes’. Analysis using the VoS viewer suggests a shift in research focus towards the correlation between osteocalcin levels and diabetic complications, the clinical efficacy of therapeutic agents or vitamins in the treatment of osteoporosis in diabetic patients, and the mechanisms by which osteocalcin modulates insulin action. The proposed focus areas are “osteocalcin genes”, “insulin regulation and osteoporosis “, “different populations”, “diabetes-related complications” and “type 2 diabetes mellitus”,“effect of osteocalcin expression on insulin sensitivity as well as secretion”,“osteocalcin expression in different populations of diabetic patients and treatment-related studies”.

## Introduction

Diabetes mellitus is a chronic metabolic disease characterised by a disorder of glucose metabolism resulting from the consistent presence of beta-cell dysfunction leading to insufficient insulin secretion, reduced insulin sensitivity, or both. In 2021, there were 529 million(95% uncertainty interval [UI] 500-564) people with diabetes globally, with an overall global age-standardised prevalence of diabetes of 6-1%(5-8-6-5), and a global age-standardised prevalence of diabetes of 6-1%(5-8 -6-5), representing a major public health challenge (1).

In addition to the most common secondary complications of diabetes including diabetic retinopathy, nephropathy, neuropathy, and cardiovascular disease (2), bone disorders are frequently diagnosed, and thus diabetes is classified as a bone-related disease. It is characterised by altered bone mineral density(BMD), abnormal bone metabolism and microarchitecture, and reduced bone strength (3).

Recent studies have suggested that osteocalcin may play an important role in regulating blood glucose levels and controlling the development of diabetes mellitus (4). Osteocalcin, also known as bone γ-carboxyglutamic acid(Gla) protein or BGP, is a 46-50 amino acid, 5.6 kDa secreted protein produced mainly by osteoblasts (5), while osteocalcin(OC) is the most abundant non-collagenous and osteoblastic bone secreted protein. It consists of carboxylated OC(cOC) and undercarboxylated OC(ucOC) forms (6).

OC, especially ucOC, can improve pancreatic function and metabolic status by targeting multiple tissues essential for glucose and lipid metabolism. In the pancreas, ucOC can directly promote β-cell proliferation and insulin production via GPRC6A (7, 8), and ucOC also indirectly promotes insulin production by increasing intestinal glucagon-like peptide-1 (GLP-1) levels and thereby promoting GPRC6A secretion (9). In insulin-targeted tissues, ucOC can increase glucose and fatty acid uptake (10), insulin sensitivity (11), nutrient utilisation and mitochondrial capacity, and reduces glycogen production in muscle and lipid synthesis in the liver (12, 13). Moreover, non-carboxylated osteocalcin ucOC is regulated by insulin and increases β-cell proliferation as well as insulin production and secretion, whereas skeletal muscle and adipose tissue respond to osteocalcin by increasing insulin sensitivity (14).

The study of the link between osteocalcin and diabetes suggests that we can control blood glucose by regulating osteocalcin levels.

A substantial body of evidence from numerous studies indicates that osteocalcin plays a role in regulating insulin secretion and in the prevention and treatment of diabetes. The numerous cross-sectional studies conducted provide a strong scientific basis and practical application for this finding.

In a comprehensive and systematic review of previous studies on osteocalcin, Meredith L. Zoch (14) and colleagues provide a new perspective on the role of osteocalcin in bone metabolism, reproduction, and cognition. They demonstrate that osteocalcin is a bone-derived factor that affects these processes through endocrine circuits in bone. This study challenges the conventional view of osteocalcin perception.

Naomi Dirckx (15) and others further reviewed and analysed the research in this field in relation to osteoblasts, reviewing the specific response of osteoblasts to metabolic hormones as well as the production of at least three endocrine factors affecting whole-body metabolism from an osteoblast perspective. Setor Kwadzo Kunutsor (16) et al. reviewed and meta-analysed serum total osteocalcin in relation to type 2 diabetes and intermediate metabolic phenotypes. Dong-Mei Liu et al. (17) outlined bone as another potential target for the treatment, prevention and prediction of diabetes. Yixuan Li (18) et al. focused on bone-derived hormones such as fibroblast growth factor 23 and osteocalcin, and summarised their new therapeutic roles in the regulation of diabetes and diabetic nephropathy. Monika Martiniakova (3) and others discussed the current role of osteocalcin in the management and treatment of diabetes mellitus, osteoporosis, osteomalacia, and inflammatory joint diseases from a preclinical as well as a clinical research perspective, and reviewed the latest disease research advances of osteocalcin.

Although the above review articles provide insights and value for understanding some specific aspects of the research on the relationship between diabetes and osteocalcin, they fail to consider the skeleton as a whole as an endocrine organ. Instead, they focus on osteocalcin as part of the narrative, examining only the mechanism of osteocalcin or the treatment of diabetes. Furthermore, these reviews do not analyse osteocalcin and diabetes in a systematic manner. They also fail to provide insights into the range of topics covered in the research area, as well as the composition and geographical distribution of influential journals and distinguished authors and institutions in this scientific field, and its history and latest trends. Furthermore, the articles in question do not provide insights into the range of topics covered by the research area, nor do they offer an overview of the scientific field in terms of influential journals, the composition of distinguished authors and institutions, or the geographical distribution. They also fail to provide information on the history and recent trends of the field. This is not a deficiency of the literature itself, but rather a consequence of the choice of focus, coverage, and research methods and tools employed in these review articles.

Bibliometrics is a comprehensive scientific mapping and analysis tool that enables the analysis and processing of vast quantities of literature information, thereby enhancing the efficiency of scientific research (19). It is employed to illustrate relationships, such as citations, collaborations, and co-occurrences, within the literature. It is also used to construct various types of knowledge graphs and to explore the critical paths, research hotspots, and cutting-edge directions of the evolution of a discipline or field. The visualisation method of co-citation analysis in bibliometrics assists in the interpretation of data and defines a relationship between two articles if they are cited by one or more other articles at the same time. Co-citation analysis allows for the assessment of the relative importance placed by researchers on the cited literature.

However, to the best of our knowledge, there are no previous bibliometric studies that have reported an association between osteocalcin and diabetes.

It was therefore our intention to address this knowledge gap. In this study, the bibliometric software Bibliometrix and the visualisation tool VOSviewer were employed to analyse the literature pertaining to osteocalcin and diabetes and to map scientific knowledge.

## Materials and methods

### Literature sources and search strategy

After obtaining relevant title keywords from PubMed and supplementing them with web subject terms, we initiated an exhaustive online bibliographic search via WoS in the following format:

All articles from October 1986 to May 2024 were searched in the Web of Science Core Collection (WoSCC) database using the search formula (ALL = (Osteocalcin)) AND ALL = (diabetes or diabetes mellitus).

A meticulous examination of the extant literature yielded 1979 potential results, which were subjected to a rigorous screening process. This yielded the registration of 1680 papers, as illustrated in

**Figure 1:**
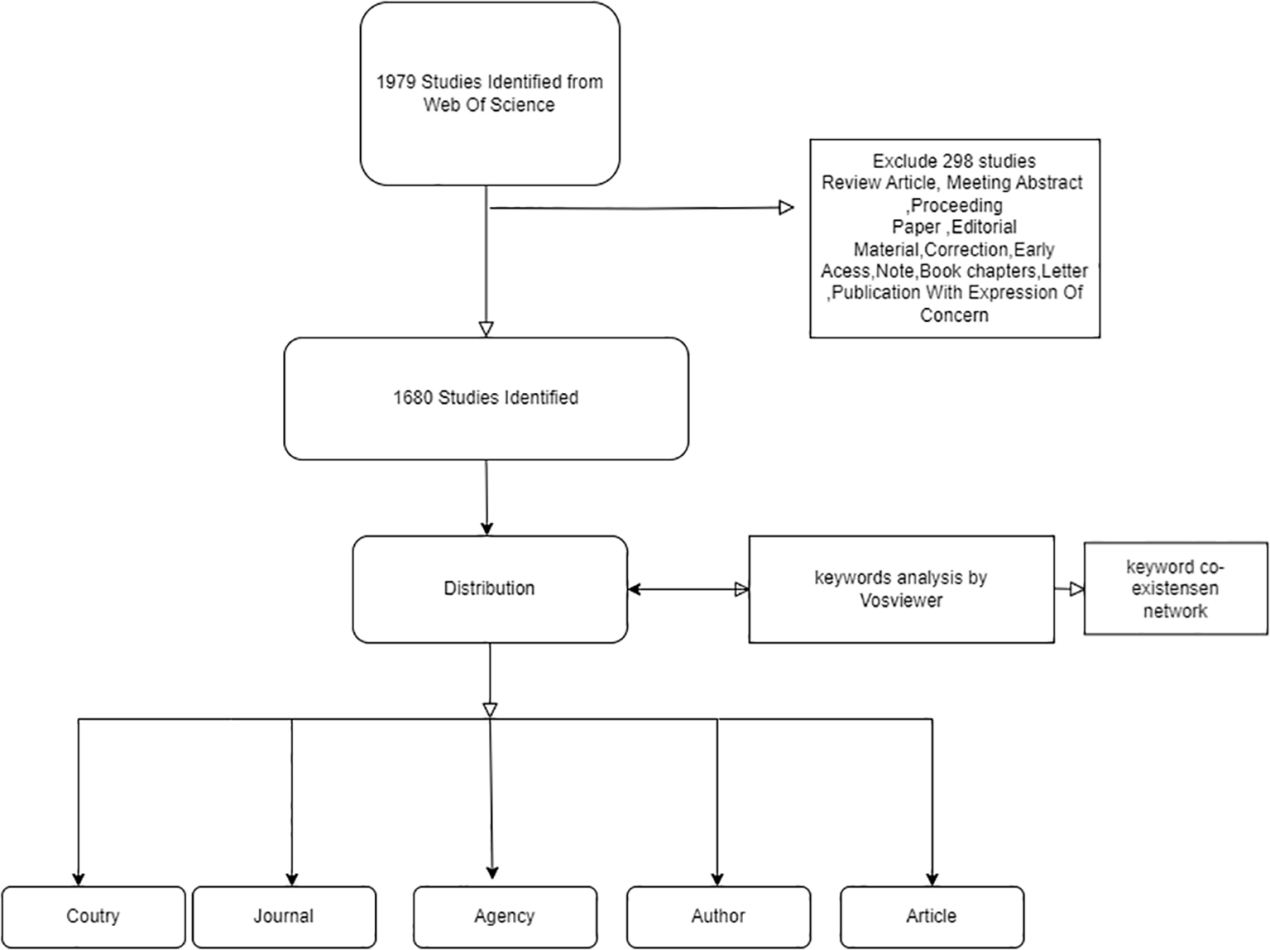
Inclusion and exclusion criteria for diabetes in osteocalcin studies.

**Figure 1**-Inclusion and exclusion criteria for diabetes in osteocalcin studies.

### Data collection and statistics

The raw data downloaded from WoSCC was initially imported into Microsoft Excel 2019 for preliminary collation. Subsequently, two researchers (FZG and WJ) conducted independent verification assessments. In the event of discrepancies, the assessment was reassessed by a third party and immediately triangulated. Finally, the bibliometric parameters were extracted, namely the number of papers and citation frequency.

The statistical methods employed included importing the collated data into the bibliometric online analysis platform (https://bibliometric.com/) for statistical analysis of the total number.

A cluster analysis of keywords was conducted using VoS viewer, based on their occurrence in titles and abstracts (19). Furthermore, the frequency and interconnections of different keywords were described by the colour, size and connecting lines of the circles (20).

## Result

### Annual scientific production

**Figure 2:**
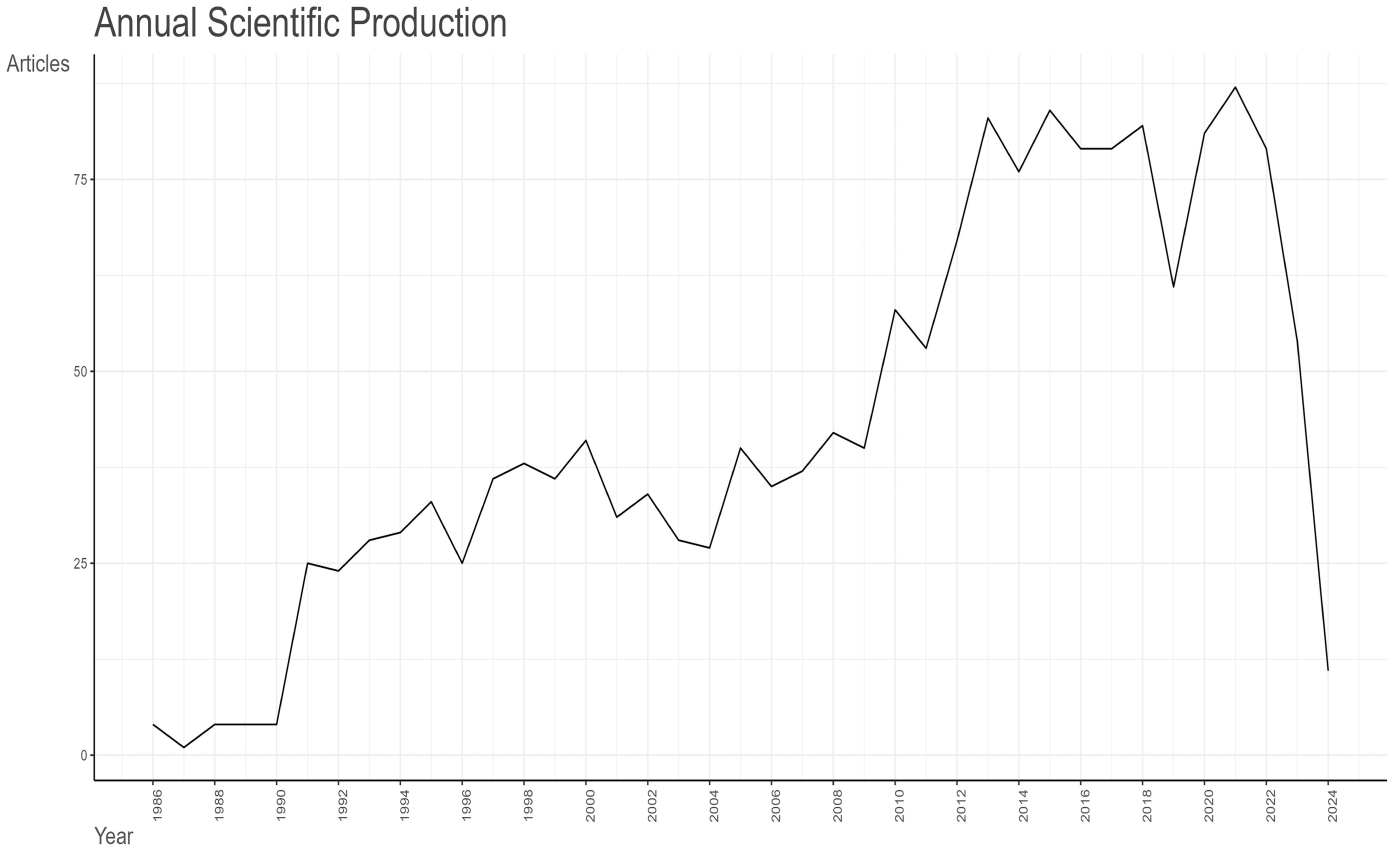
Line graph of the number of articles published over time.

As illustrated in **Figure 2** **-** Line graph of the number of articles published over time., the earliest pertinent article was published in 1986. Between 1986 and 1990, the field is still in its infancy, with no more than five articles published each year. In 1991, there was a notable increase in the number of articles published, which can be attributed to the advancement of molecular biology techniques, such as the study of the osteocalcin gene sequence (21). This resulted in a scientific output that was greater than that of the previous five years combined. The period from 1991 to 2013 demonstrates a consistent upward trajectory, punctuated by fluctuations. The greatest increase in articles occurred in 2013, due to the proposal that “research was needed to define the role of osteocalcin and its carboxylated or under-carboxylated forms in the regulation of human glucose metabolism” (22). Consequently, as a consequence of the evolution of research themes, the annual scientific production of articles shows a decreasing trend, with two notable declines occurring in 2019 and 2023. In 2019, the majority of studies were related to osteoporosis, a complication of diabetes mellitus. The data from 2023 and 2024 are not relevant to the research topic due to the timing of the entry.

### Average number of citations per year

**Figure 3:**
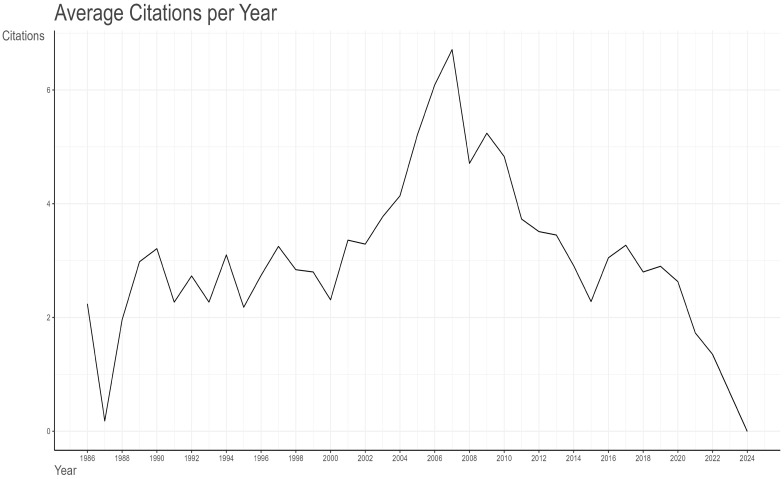
A line graph showing the total citation volume per article over time.

A total of 81,674 citations have been made of the 1,680 articles selected from the Web of Science (WoS) database, with an average citation frequency of 48.62 per article. As illustrated in **Figure 3** **-** A line graph showing the total citation volume per article over time., the peak of the average total citations per article was observed in 2007, with 120.76 citations per article as of 19 May 2024. This was followed by 2006, with 115.63 citations per article. Further analysis of the graph of the average number of citations per article per year in **Table 1** Since 1986, the number of articles and citation counts in this field(MeanTCperArt means the mean number of total citations per article. N means the total number of articles in that year. MeanTCperYear means the mean number of MeanTCperArt per article) reveals that 2007 was the most prolific year, with an average of 6.71 citations per article per year. This was followed by 2006 (6.09), 2009 (5.24), and 2005 (5.21). 2010 ranked fifth, with an average of 4.83 citations per article per year and a total of 4,190 citations. In 2010, the literature was ranked fifth, with an average of 4.83 citations per article per year and a total of 4,199 citations, which placed it second only to the 4,468 citations in 2007. A detailed examination of these three types of data reveals the intricate nature of the influence of scholarly outputs and underscores the significance of a comprehensive assessment of the impact of the scholarly literature.

**Table 1:** Since 1986, the number of articles and citation counts in this field (MeanTCperArt means the mean number of total citations per article.

Year	MeanTCperArt	N	MeanTCperYear	TC
1986	87.25	4	2.24	349
1987	7.00	1	0.18	7
1988	72.50	4	1.96	290
1989	107.25	4	2.98	429
1990	112.50	4	3.21	450
1991	77.24	25	2.27	1931
1992	90.25	24	2.73	2166
1993	72.50	28	2.27	2030
1994	95.97	29	3.10	2783
1995	65.39	33	2.18	2158
1996	79.60	25	2.74	1990
1997	90.97	36	3.25	3275
1998	76.74	38	2.84	2916
1999	72.67	36	2.80	2616
2000	57.68	41	2.31	2365
2001	80.61	31	3.36	2499
2002	75.76	34	3.29	2576
2003	83.00	28	3.77	2324
2004	86.93	27	4.14	2347
2005	104.20	40	5.21	4168
2006	115.63	35	6.09	4047
2007	120.76	37	6.71	4468
2008	80.14	42	4.71	3366
2009	83.80	40	5.24	3352
2010	72.40	58	4.83	4199
2011	52.21	53	3.73	2767
2012	45.61	67	3.51	3056
2013	41.43	83	3.45	3439
2014	32.03	76	2.91	2434
2015	22.80	84	2.28	1915
2016	27.44	79	3.05	2168
2017	26.14	79	3.27	2065
2018	19.59	82	2.80	1606
2019	17.43	61	2.90	1063
2020	13.17	81	2.63	1067
2021	6.90	87	1.73	600
2022	4.05	79	1.35	320
2023	1.33	54	0.66	72
2024	0.00	11	0.00	0

N means the total number of articles in that year.

MeanTCperYear means the mean number of MeanTCperArt per article.

### Quantity and citations among different nations

**Figure 4:**
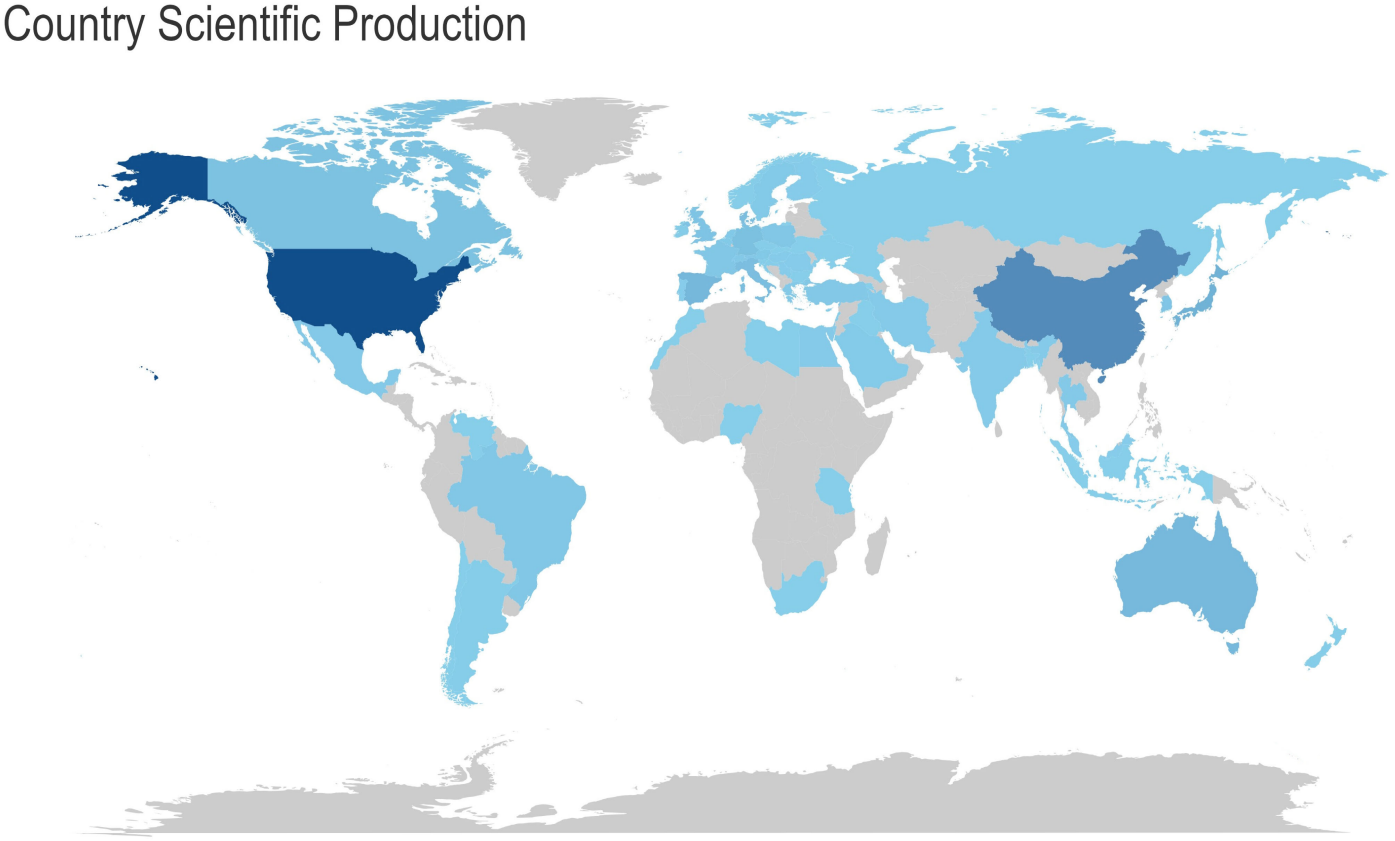
Analysis of research output in the field by various countries. The colour of the country represents the number of publications in that field. The darker the colour, the greater the number of articles published in that field.

**Figure 4** Analysis of research output in the field by various countries. The colour of the country represents the number of publications in that field. The darker the colour, the greater the number of articles published in that field. provides a global overview of the volume of scientific output in this field, with the United States accounting for the largest number of publications in this field. **Table 2** **-** Top 10 Countries with the Highest Productivity presents a comparative analysis of the performance of different countries in terms of scientific research output, based on three sets of data. The countries included in the analysis were selected based on their ranking in both the Countries’ Scientific Production and Most Cited Countries categories. The United States, with 2,411 scientific production articles, a total of 45,359 citations and an average citation rate of 81.7 citations, was at the top of the list, clearly outperforming China and Japan. China’s scientific production was 1,198 articles, with a cumulative total of 4,444 citations and an average citation rate of 16.5 citations. Japan’s scientific production amounted to 501 articles, with a cumulative total of 4,810 citations and an average citation rate of 37.6 citations, which is more than double that of China. The article with the highest number of citations in Japan was that which concluded that “osteocalcin was important not only for bone metabolism, but also for glucose and fat metabolism” (23). This was achieved by studying the parameters of atherosclerosis in men with type 2 diabetes mellitus and postmenopausal women. The highest number of citations for a single article in China was 198, and the article (24) concludes that “mucins could mimic the biological effects of insulin”.

**Table 2:** Top 10 countries with the highest productivity.

Region	Countries’ Scientific Production	Most Cited Countries	Average Article Citations
USA	2411	45359	81.7
CHINA	1198	4444	16.5
JAPAN	501	4810	37.6
SPAIN	389	2027	37.50
AUSTRALIA	370	1880	38.4
ITALY	275	1731	31.5
DENMARK	273	1210	31.8
GERMANY	233	1727	38.4

**Figure 5:**
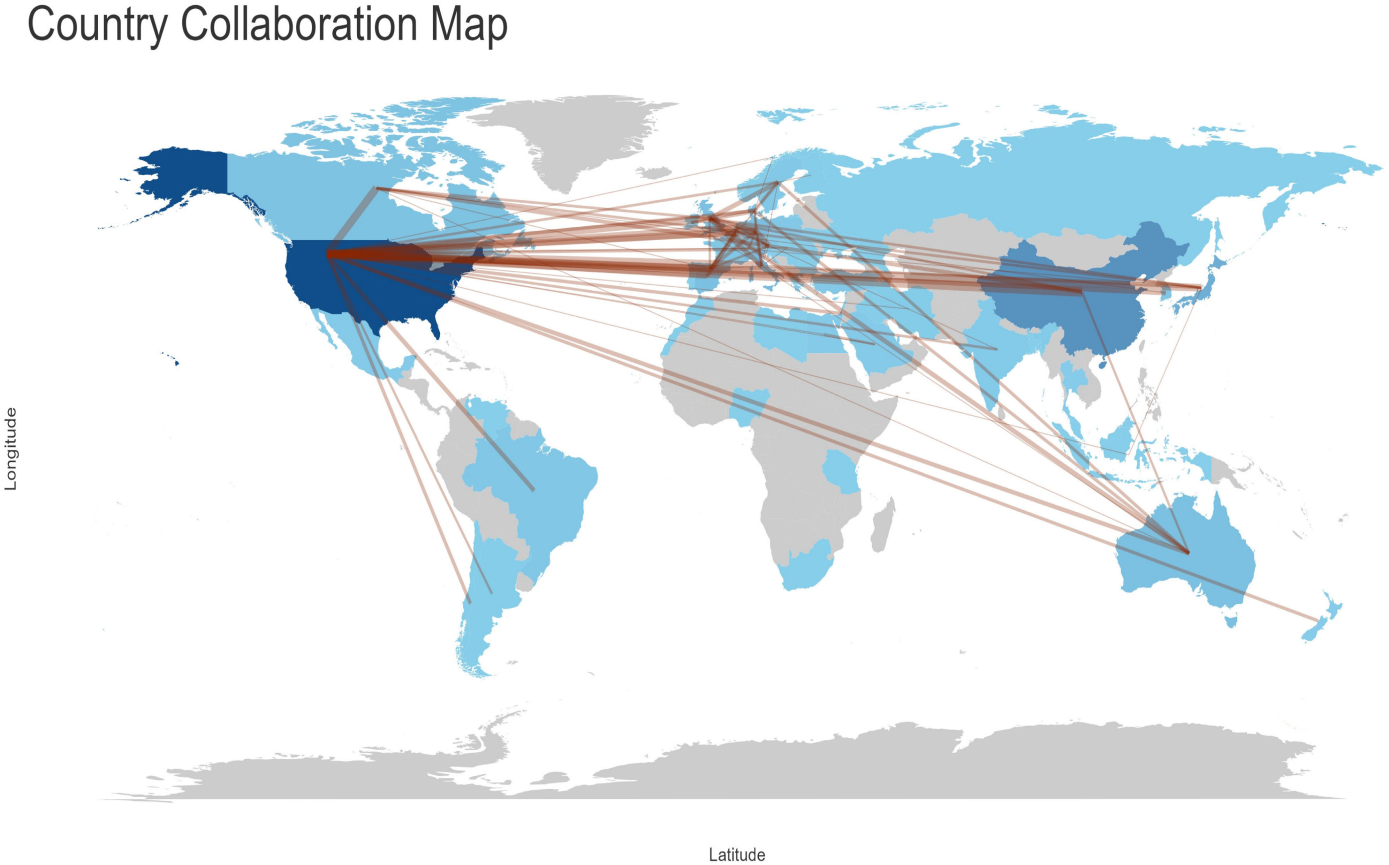
Analysis of collaboration objectives among nations, with lines indicating cooperation projects between two countries.

As illustrated in **Figure 5** **-** Analysis of collaboration objectives among nations, with lines indicating cooperation projects between two countries, the United States had the greatest number of interrelated targets (36 countries, including China, Italy, Canada, the United Kingdom, Japan, Germany, France, and others) in terms of country cooperation. It is recommended that efforts be intensified to reinforce international cooperation and communication.

### High contribution journals

The top 10 journals by publication volume collectively published 576 articles, representing 34.29% of the total (**Table 3** **-** Top 10 Relevant Popular Journals). Among the journals, the Journal of Clinical Endocrinology & Metabolism had the highest number of publications (105 articles) and the most total articles published (89,559). Meanwhile, the Journal of Bone and Mineral Research had the highest Journal Citation Indicator (JCI) (1.38) and Impact Factor (6.2).

**Table 3:** Top 10 relevant popular journals.

Sources	N	%	Total Citations	JCI	IF-2022	JCR
JOURNAL OF CLINICAL ENDOCRINOLOGY & METABOLISM	105	6.25	89,559	1.27	5.8	Q1
JOURNAL OF BONE AND MINERAL RESEARCH	89	5.30	29,005	1.38	6.2	Q1
BONE	87	5.18	25,618	1.01	4.1	Q2
ENDOCRINOLOGY	70	4.17	41.684	0.89	4.9	Q2
OSTEOPOROSIS INTERNATIONAL	57	3.39	20,508	0.93	4	Q2
CALCIFIED TISSUE INTERNATIONAL	45	2.68	8,718	0.81	4.2	Q2
JOURNAL OF BIOLOGICAL CHEMISTRY	45	2.68	336,186	0.87	4.8	Q2
CLINICAL ENDOCRINOLOGY	28	1.67	15,283	0.66	3.2	Q3
MOLECULAR ENDOCRINOLOGY	26	1.55	11,646	–	3.628	Q2
PLOS ONE	24	1.43	886,919	0.91	3.7	Q2

### The number of articles published by different institutions

**Figure 6:**
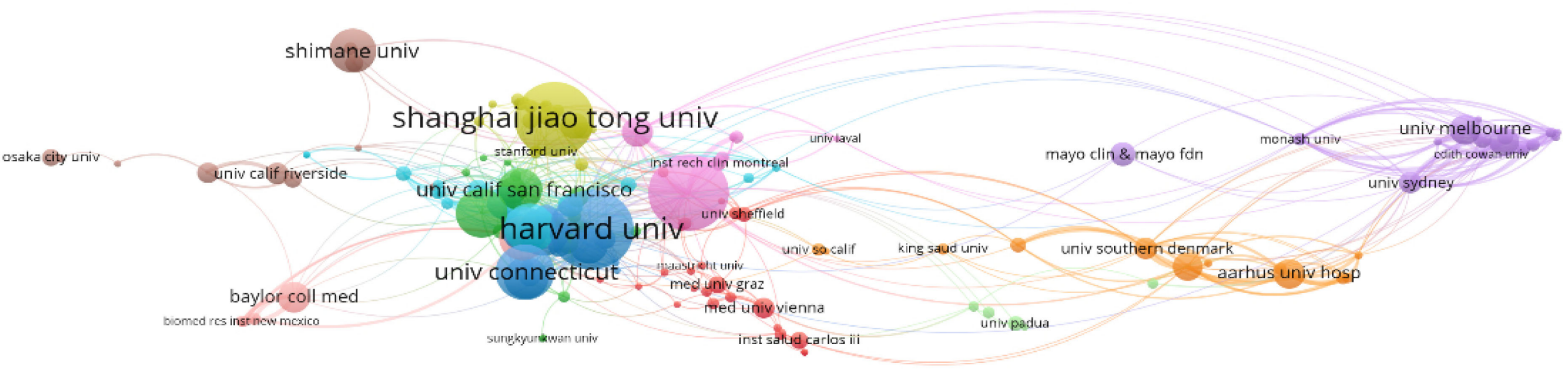
Statistical analysis of the number of articles published by institutions. The larger the cluster, the more articles published.

In terms of the number of publications, Harvard University in the United States is the leading institution, with 53 articles, as illustrated in **Figure 6** **-** Statistical analysis of the number of articles published by institutions. The larger the cluster, the more articles. Columbia University follows with 51 articles. The third-ranked institution is Shanghai Jiao Tong University from China, with 50 articles, which also represents the highest number of publications from a single institution in China. Among the top ten research institutions, eight are from the United States, with the remaining two being Shanghai Jiao Tong University from China and Shimane University from Japan.

### Collaboration network

**Figure 7:**
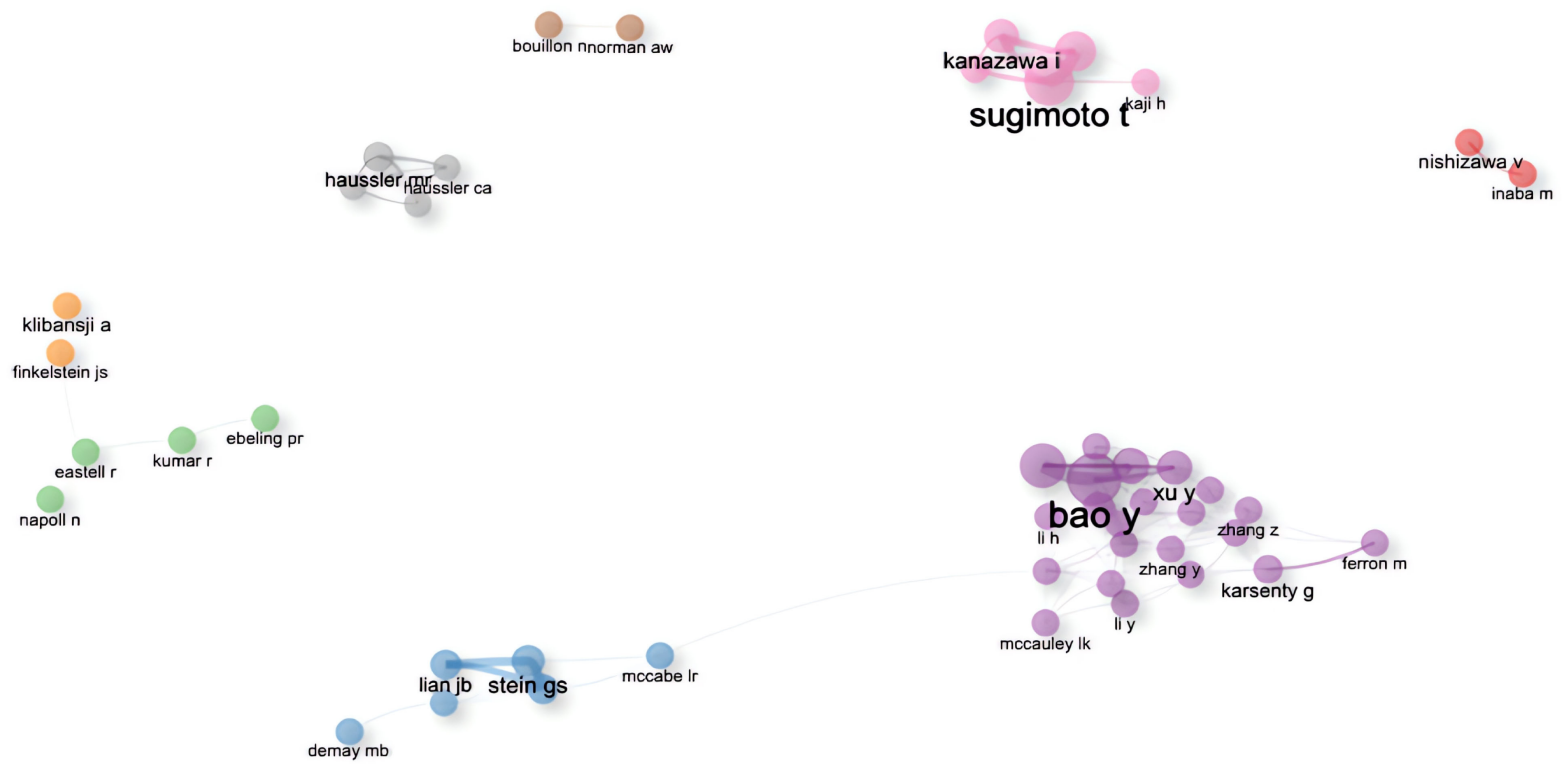
Collaborative relationships among authors. Divided into 8 clusters, the connections between nodes indicate the existence of collaboration, with the thickness of the lines representing the strength or frequency of the collaboration. The author names at the centre of each cluster represent the key Figures of that cluster.

The author collaboration network graph generated by Bibliometrix has eight clusters after excluding individual authors as clusters. The first cluster is purple, the second is blue, the third is pink and the fourth is grey. The first group consisted mainly of Chinese researchers from Shanghai Jiao Tong University (SJTU), and its core Figure was Prof. Yuxian Bao, who had the strongest collaborative relationship among the four of them, namely Academician Weiping Jia, Prof. Xiaojing Ma and Scholar Yiting Xu. It should be noted that the graph shows that the scholar in the first group, Jing Zhang, had made contact with the second group, but subsequent checking of the information revealed that the researcher working with Prof Bao in the first group was from SJTU, and the researcher working with L R McCabe in the second group was from Michigan State University. The names of these two people are repeated, resulting in an assignment error. Meanwhile, the first group shown in **Figure 7**-Collaborative relationships among authors. Divided into 8 clusters, the connections between nodes indicate the existence of collaboration, with the thickness of the lines representing the strength or frequency of the collaboration. The author names at the centre of each cluster represent the key Figures of that cluster. worked closely with the fourth group. However, due to the abbreviation of the names, Xinmei Zhang collaborated with Prof. Levinger, Itamar in group 4, and Xiaohui Zhang collaborated with Prof. liu y, neither of whom was from SJTU. The liu y in the fourth group also refers to different people, which does not indicate that the first and fourth groups worked closely together. The second group was led by Prof. Stein, Gary S. from the University of Vermont, and had close cooperation with Prof. Lian, Jane B. and Prof. Stein, Janet L. The members of this group came from different research institutions in the United States, such as the University of Vermont, the University of Massachusetts, Harvard University, the University of Wisconsin, and Michigan State University, and so on. In **Figure 7**-Collaborative relationships among authors. Divided into 8 clusters, the connections between nodes indicate the existence of collaboration, with the thickness of the lines representing the strength or frequency of the collaboration. The author names at the centre of each cluster represent the key Figures of that cluster., the members of the second group worked closely with the third group. In fact, it is Prof. Yamauchi, Miki, who had collaborated with the members of the second group, not Yamauchi, Mika, who was the researcher of the third group, and the central Figure of the third group was Prof. Sugimoto, Toshitsugu of Shimane University, who had collaborated with Prof. Kanazawa, Ippei, Prof. Yamauchi, Mika, and Prof. Yamaguchi, Mika. Professor Sugimoto, Toshitsugu of Shimane University, who had a strong collaborative relationship with Professor Kanazawa, Ippei, Professor Yamauchi, Mika and Professor Yamaguchi, Toru, and the four of them had a total of seven articles related to the topic of this paper on the Web of Science, compared with the four in the first group who had a total of eight articles related to the topic of this paper.

**Figure 8:**
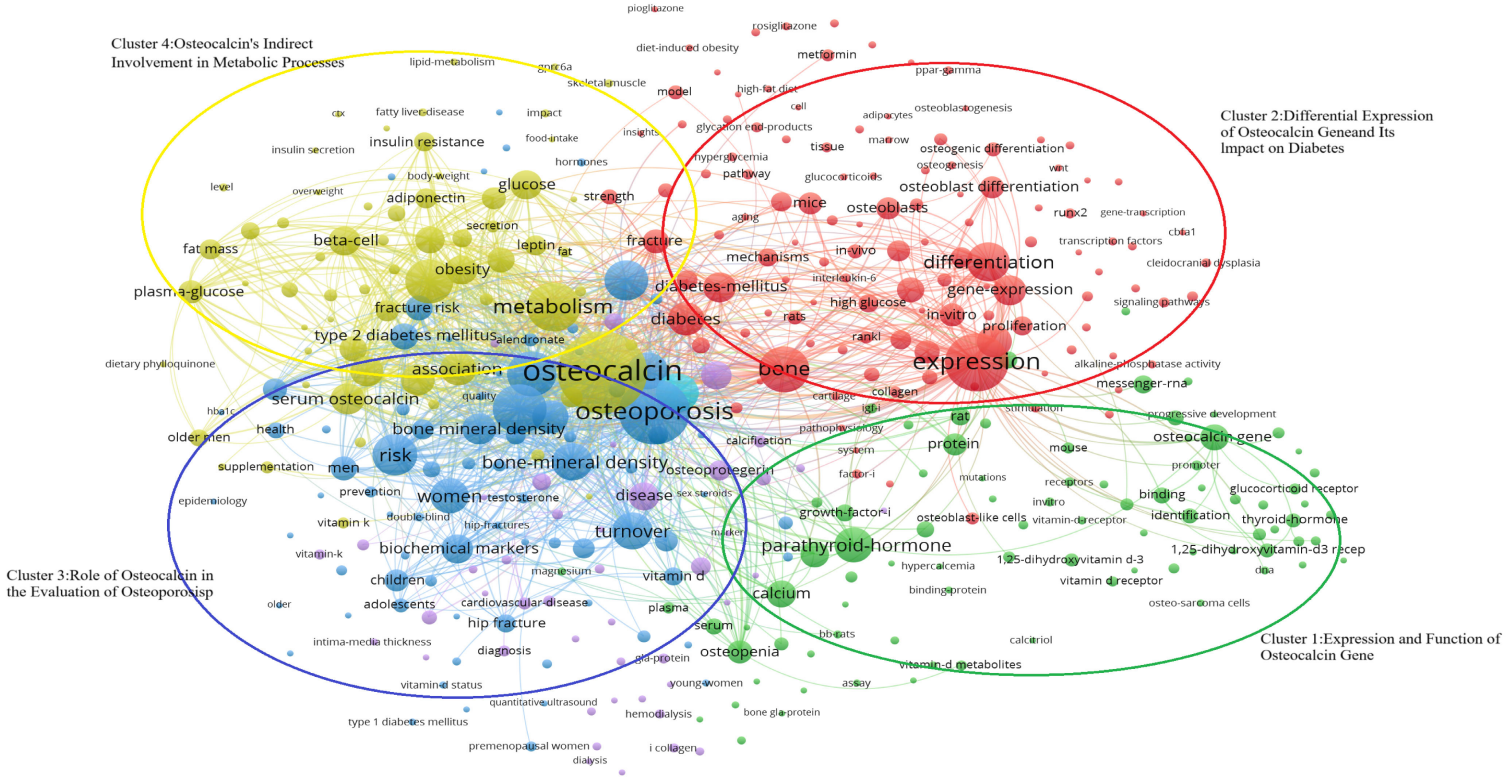
Keywords scientific analysis clustering. (Cluster 1:Expression and Function of Osteocalcin Gene; Cluster 2:Differential Expression of Osteocalcin gene and Its Impact on Diabetes; Cluster 3;Role of Osteocalcin in the Evaluation of Osteoporosis; Cluster 4:Expression and function of Osteocalcin’s Indirect Involvement in Metabolic Processes).

Keywords have been filtered from the existing data, resulting in 395 words, which can be divided into four clusters, as it is shown in the **Figure 8**-Keywords scientific analysis Cluster 1, “Expression and Function of the Osteocalcin Gene,” is represented by green. Cluster 2, “Differential Expression of the Osteocalcin Gene and Its Impact on Diabetes,” is represented by blue. Cluster 3, “Role of Osteocalcin in Bone Turnover,” is represented by red. Cluster 4, “Indirect Involvement of Osteocalcin in Metabolic Processes,” is represented by yellow. The size of the keyword circles within each cluster corresponds to their frequency of occurrence.

The main terms constituting cluster 1 are “osteocalcin gene” (25, 26), “protein” (27, 28), “parathyroid hormone” (29, 30) and “1,25-dihydroxyvitamin-D3 receptor” (29, 31). Meanwhile, cluster 2 is primarily characterized by “expression” (25, 26), “difference” (25, 32), “diabetes” (33, 34) and “osteoblast” (31, 35). Cluster 3 focuses on keywords such as “osteoporosis” (36, 37), “bone turnover” (38, 39), “male” (40, 41), and “female” (42, 43). Lastly, cluster 4 includes keywords like “osteocalcin” (37, 44), “metabolic syndrome” (45, 46), “insulin resistance” (33, 47), and “plasma glucose” (41, 44).

VoS viewer marks keywords in the graph with different colours based on the average year of occurrence, with purple keywords appearing earlier than blue and yellow ones. The transition from purple to green and finally to yellow illustrates the developmental process of the keywords. The graph provides a comprehensive overview of the researchers’ investigation focus, including four clusters of keywords such as “Expression and Function of Osteocalcin Gene,” “Differential Expression of Osteocalcin Gene and Its Impact on Diabetes,” “ Role of Osteocalcin in the Evaluation of Osteoporosis and Diabetes” and “Osteocalcin’s Indirect Involvement in Metabolic Processes.”

**Figure 9:**
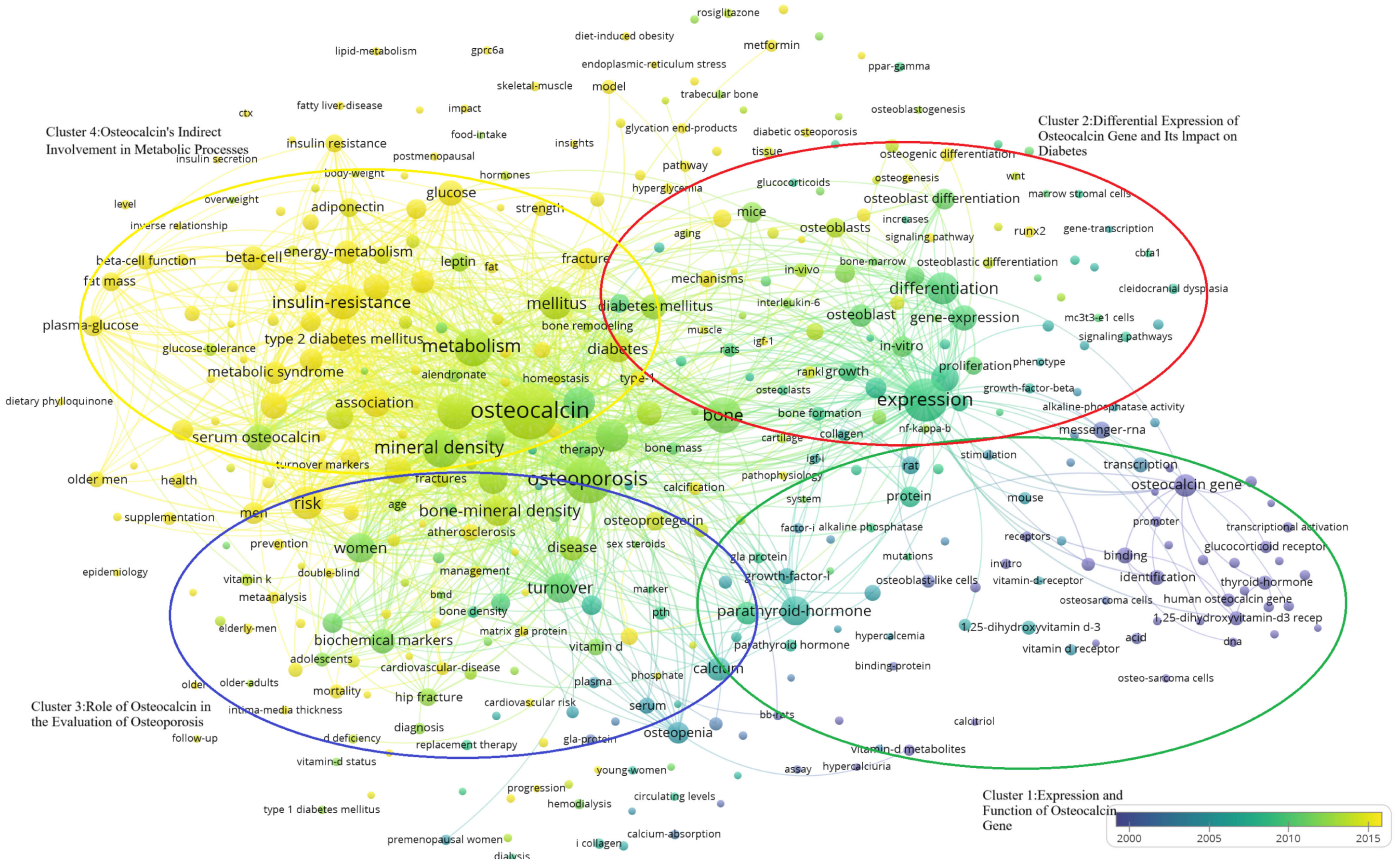
Temporal distribution of the four keyword clusters. (Cluster 1:Expression and Function of Osteocalcin Gene; Cluster 2:Differential Expression of Osteocalcin gene and Its Impact on Diabetes; Cluster 3;Role of Osteocalcin in the Evaluation of Osteoporosis; Cluster 4:Expression and function of Osteocalcin’s Indirect Involvement in Metabolic Processes).

Through bibliometrics, one can quantitatively and systematically assess trends in a specific field and predict potential research directions, as it is shown in the **Figure 9** **-**. In this study, keywords were categorized into four clusters:

“Expression and Function of Osteocalcin Gene”(Cluster 1, Green) “Differential Expression of Osteocalcin Gene and Its Impact on Diabetes”(Cluster 2, Blue) “ Role of Osteocalcin in the Evaluation of Osteoporosis and Diabetes”(Cluster 3, Red) “Osteocalcin’s Indirect Involvement in Metabolic Processes”(Cluster 4, Yellow). In the **Figure 9** **-**, it shows that the researchers’ investigative interests shifted from Cluster 1 to Cluster 2, then to Cluster 3, and finally to Cluster 4.

### Characteristics of the top 10 most cited research articles

**Figure 10:**
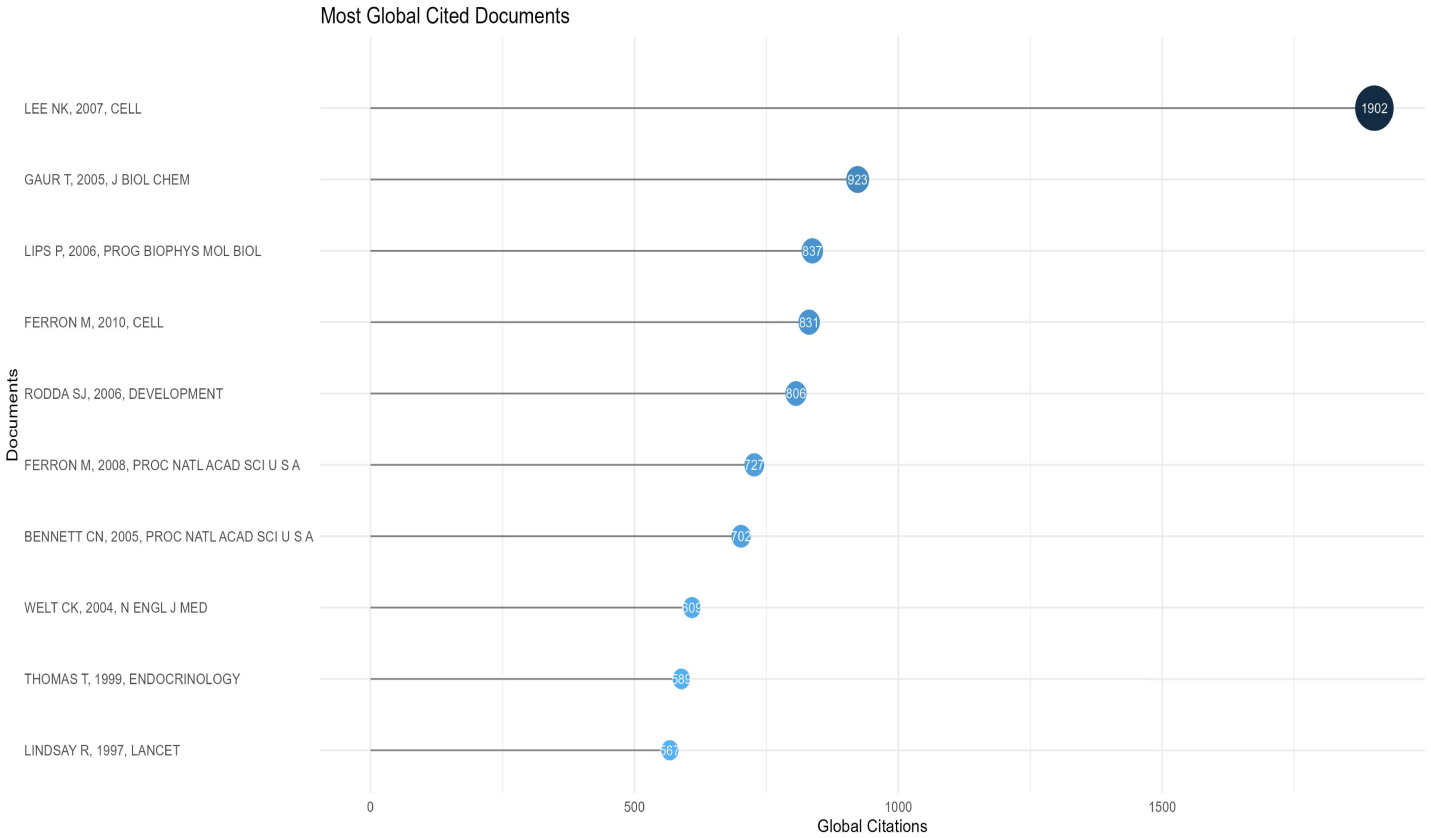
Top 10 most global cited articles.

The 10 most cited articles were cited a total of 8493 times (10.40%, Temporal distribution of the four keyword clusters. (Cluster 1:Expression and Function of Osteocalcin Gene; Cluster 2:Differential Expression of Osteocalcin gene and Its Impact on Diabetes; Cluster 3;Role of Osteocalcin in the Evaluation of Osteoporosis; Cluster 4:Expression and function of Osteocalcin’s Indirect Involvement in Metabolic Processes) (**Figure 10**- Top 10). The third most cited article was a review, and Prof. Na Kyung Lee’s 2007 article (48) ranked first in both total citations and citation frequency per year, with 1902 and 105.67, respectively. This article accounted for 42.6% of the total citations in 2007. In 2005, articles by Tripti Gaur (26) and Christina N Bennett (28) ranked second and seventh in total citations, with 923 and 702, respectively. These two articles combined account for 38.99% of the citations for the year. Mathieu Ferron’s articles in 2010 (27) and 2008 (25) ranked fourth and sixth at 831 and 721, respectively, accounting for 19.79% and 21.42% of that year’s articles. His two articles accounted for 18.27% of the 10 most cited articles. Thus, the average number of citations per year is determined by a combination of annual scientific output and high-impact articles, with the latter playing a more pronounced role. The ten articles are presented in **Table 4**-Top 10 Most Cited Research Paper for the reader’s convenience.

**Table 4:** Top 10 most cited research paper.

No	First author	Journal	Year	Citations	Citation frequency per year	Descriptions
1	Na Kyung Lee (48)	CELL	2007	1902	105.67	Studies have shown that mice deficient in osteocalcin develop reduced β-cell proliferation, glucose intolerance and insulin resistance, reflecting the function of osteocalcin in improving glucose tolerance *in vivo* and demonstrating, for the first time, that the skeleton has an endocrine regulatory role in energy metabolism.
2	Tripti Gaur (26)	J BIOL CHEM	2005	923	46.15	In SFRP1-null mice, canonical WNT signaling directly upregulates Runx2 expression in mesenchymal stromal cells, making it a target of β- Catenin/TCF1 to stimulate bone formation, promote osteocalcin secretion, and achieve control of osteoblast differentiation and skeletal development.
3	P Lips (29)	PROG BIOPHYS MOL BIOL	2006	837	44.05	The active metabolite 1,25(OH)2D promotes cellular activities such as calcium uptake and osteocalcin secretion through vitamin D receptors and produces rapid non-genomic effects through membrane receptors, which helps prevent autoimmune diseases such as type 1 diabetes and cancer.
4	Mathieu Ferron (27)	CELL	2010	831	55.40	Insulin signaling in osteoblasts plays a key role between bone remodeling and energy metabolism, and its activity influences the carboxylation state and function of osteocalcin secreted by osteoblasts. In an acidic environment, bone resorption drives protein decarboxylation, whereas insulin signaling in osteoblasts activates osteocalcin, thereby promoting glucose metabolism.
5	Stephen J Rodda (49)	DEVELOPMENT	2006	806	42.42	During differentiation of the osteoblast lineage, Ihh signaling promotes progenitor differentiation to Runx2+ precursors at an early stage, followed by Wnt/β-catenin signaling that supports their maturation into osteocalcin+ cells and prevents transformation to chondrocytes.
6	Mathieu Ferron (25)	PROC NATL ACAD SCI USA	2008	727	42.76	In WT mice, small amounts (picomolar amounts) of osteocalcin regulated insulin gene expression and beta cell proliferation, while higher concentrations (nanomolar amounts) affected protein expression in adipocytes. In addition, long-term osteocalcin treatment attenuated metabolic damage induced by a high-fat diet and gold thioglucose.
7	Christina N Bennett (28)	PROC NATL ACAD SCI USA	2005	702	35.10	Wnt10b was confirmed to be an endogenous regulator of bone formation by comparing increased bone mass and bone strength in FABP4-Wnt10b mice with decreased trabecular mass and decreased serum osteocalcin in Wnt10b-/- mice.
8	Corrine K Welt (34)	N ENGL J MED	2004	609	29.00	Leptin secreted by adipocytes serves as a peripheral signal reflecting the adequacy of energy stores and is essential for normal reproductive and neuroendocrine function, improving indicators of the reproductive, thyroid and growth hormone axes as well as bone formation (e.g., alkaline phosphatase and osteocalcin).
9	Thomas, T (35)	ENDOCRINOLOGY	1999	589	22.65	Biochemical experiments showed that leptin did not affect the proliferation of hMS2-12 in human bone marrow stromal cells, but increased the expression of bone differentiation markers (e.g., alkaline phosphatase, type I collagen, and osteocalcin), while decreasing the expression of adipocyte markers (e.g., epinephrine and leptin) and the formation of lipid droplets, suggesting that leptin promotes osteoblast differentiation and inhibits adipocyte differentiation.
10	Lindsay, R (30)	LANCET	1997	567	20.25	This trial found that 1-34 human parathyroid hormone (hPTH [1-34]) significantly increased bone mass in postmenopausal women with osteoporosis receiving hormone replacement therapy and increased osteocalcin levels by more than 55% early in the course of its use, supporting its status as a medication with a significant osteogenic effect that reduces the risk of vertebral fracture, and suggesting its potential use in the treatment of postmenopausal osteoporosis.

## Discussion

### Expression and function of osteocalcin gene

As shown in **Figure 8**-Keywords scientific analysis In cluster 1, the main terms are “osteocalcin gene”, “protein”, “parathyroid hormone”, “thyroid hormones” and “1,25-dihydroxy vitamin-D3 receptor”. This group of terms is summarized as the physiological mechanism where osteocalcin interacts with other hormones.

Osteocalcin exerts an influence on the absorption and regulation of calcium, operating in conjunction with other hormones. It has been demonstrated that thyroid hormone (TSH) exerts a pivotal influence on bone growth and development (50). The thyroid glands secrete substantial quantities of thyroxine (T4) and 3,5,3′-triiodothyronine (T3). Although the circulating levels of T3 are considerably lower than those of T4, T3 activates T3 receptors (TRα and TRβ) on osteoblasts, thereby stimulating osteocalcin secretion (51). Further investigation has revealed that TRα is the predominant isoform expressed in bone tissue, while TRβ deficiency results in a hyperthyroid effect in osteoblasts (52). Although T4 does not have as strong an affinity for the receptor as T3 (53), it has also been demonstrated in experimental studies that its osteoblastic differentiation can have a stimulatory effect (51). Furthermore, T4 and T3 have been demonstrated to directly stimulate bone resorption *in vitro* (54). Furthermore, one study indicates that estrogen status may influence the TSH response to bone (55), which could potentially elucidate the elevated prevalence of osteoporosis observed in postmenopausal women. As a consequence of TSH suppression and elevated FT4 and/or free triiodothyronine (FT3) levels in hyperthyroid patients, there is an increased rate of bone conversion, a reduction in bone mineral density (BMD), and an elevated risk of fragility fractures (56). A controlled trial of a cohort of patients with hyperthyroidism, hypothyroidism, and euthyroidism revealed that blood osteocalcin levels were markedly elevated in the hyperthyroid group and markedly reduced in the hypothyroid group when compared to the euthyroid control group (57). In patients with hyperthyroidism, antithyroid hormone medication has been demonstrated to reduce blood osteocalcin concentrations. Conversely, in patients with hypothyroidism, L-T4 has been shown to be an effective means of restoring osteocalcin levels (58). It has been demonstrated that thyroid hormone can facilitate osteocalcin synthesis in osteoblasts by activating p38 MAP kinase (59). Consequently, the thyroid hormone plays a pivotal role in regulating bone function, and influencing bone metabolism. Hyperthyroidism is a recognized cause of secondary osteoporosis (60), which is characterized by accelerated bone turnover and a negative calcium and phosphorus balance (61). In addition to its role in bone conversion and formation, the promotion of osteocalcin synthesis, which leads to increased bone conversion, may also be a pathway by which hyperthyroidism causes osteoporosis.

With regard to parathyroid hormone (PTH), it is once more an essential peptide hormone that regulates bone formation and osteoblast activity. Parathyroid hormone is capable of regulating extracellular calcium and phosphorus levels and stimulating bone resorption. Two principal pathways are responsible for the regulatory effects of parathyroid hormone on bone: the PKA pathway and the PKC pathway. The regulatory effects of parathyroid hormone on bone are mediated by direct activation of parathyroid hormone receptor 1 (PTHR1) on osteoblasts, which stimulates Gα_s_-mediated activation of adenylate cyclase and promotes the production of cAMP, followed by the activation of protein kinase (PKA) (62). Subsequently, the activation of the Raf-MEK-ERK mitogen-activated protein kinase (MAPK) cascade reaction (63) results in a proliferative effect on bone (64). Furthermore, the production of cAMP and the activation of PKA can also activate G_q/11_-mediated PLCβ stimulation, which in turn leads to the production of inositol 1,4,5-trisphosphate (IP_3_), the mobilization of calcium and the activation of protein kinase C (PKC) (65). PKC then activates MAP kinase and exogenous signal-regulated kinase (ERK), which in turn results in the onset of osteoblast proliferation (64). Furthermore, it has been demonstrated that parathyroid hormone can act synergistically through the PKA and PKC pathways to co-stimulate osteocalcin gene expression in osteoblasts (66). This pathway directly activates osteoblast OCN gene transcription by facilitating the binding of the OSE1 sequence proximal to the mOG2 promoter to nuclear proteins (67). Clinical trials have demonstrated that increased parathyroid hormone levels lead to elevated osteocalcin levels (68) and are effective in ameliorating idiopathic osteoporosis in men (69). Notably, several experiments have shown that both instantaneous and intermittent administration of parathyroid hormone are effective in stimulating bone formation and repair (70, 71), which appears to be related to the gradient sensitivity of parathyroid hormone (71).

OSE2 is a specific cis-acting element present in osteoblasts, and Osf2/Cbfa1 is a protein that binds to OSE2, regulated by Bone Morphogenetic Protein 7(BMP7) and Vitamin D-3. Osf2/Cbfa1 binds to and regulates the expression of multiple genes in osteoblasts (72). Research indicates that 1,25D regulates bone mineral remodelling by inducing Receptor Activator of Nuclear Factor κB Ligand(RANKL), Secreted Phosphoprotein 1 Gene(SPP1), and Bone Gla Protein(BGP), and also controls calcium and phosphorus reabsorption in the kidneys, with the skeleton acting as a sensor for phosphorus levels (73). Upon binding with the Vitamin D receptor, 1,25-dihydroxyvitamin D3(1,25D) directly inhibits parathyroid hormone gene transcription and antagonizes osteocalcin to prevent hypercalcemia (29). Controlling osteocalcin expression may delay age-related chronic diseases such as osteoporosis, type 2 diabetes, cardiovascular disease, and cancer.

While not directly involved in bone mineralization, osteocalcin has been identified as a factor that plays a role in preventing excessive bone mineralization and increased bone fragility in osteoporosis. The absence of osteocalcin in experimental animals led to the observation that, despite the normal survival of osteocalcin-free mutant mice, their cortical bone width was significantly higher than that of the wild type after six months. This was observed in the absence of an increase in osteoclast number, mineralization rate, and bone mineral content (74). This indicates that osteocalcin is not a factor in the process of mineralization. Subsequently, the researchers induced osteoporosis in the mutant and wild-type mice by removing their ovaries. This resulted in more pronounced osteoporosis symptoms in the mutant mice than in the wild-type, which may be attributed to an increased number of osteoclasts in the mutant mice. This indicates that osteocalcin exerts its effect by limiting bone matrix formation, without influencing resorption or mineralization. The relative amount of hydroxyapatite phosphate is diminished in osteocalcin-deficient mice, which exhibit reduced bone maturation (75), indicating that osteocalcin deficiency may be associated with increased bone fragility. It may therefore be posited that the administration of vitamin K (76), 1,25-dihydroxy-D3 (77) to patients exhibiting low osteocalcin levels may prove an efficacious method of reducing the risk of osteoporosis, particularly in those already diagnosed with the condition. In the context of long-term warfarin anticoagulation, the potential for reducing the risk of osteoporosis may be realized through the replacement of warfarin with a non-vitamin K-dependent anticoagulant, such as dabigatran (78).

The association between osteocalcin levels and type 2 diabetes has been demonstrated in numerous studies across diverse populations (36, 79, 80). In animal experiments, it has been demonstrated that osteocalcin is associated with the regulation of blood glucose levels and improved insulin sensitivity and secretion (48). Intermittent osteocalcin injections have been shown to increase glucose tolerance and insulin sensitivity, improve pancreatic β-cell mass, and prevent hepatic steatosis in mice (44). While this treatment regimen has not yet been implemented in clinical practice (81), it represents a promising avenue for future diabetes treatment and prevention strategies.

Despite evidence from statistical experiments indicating a potential correlation between osteocalcin and cardiovascular disease (82–85), the precise mechanism of action remains unclear. Osteocalcin has been shown to exert protective effects on the vascular endothelium in both animal (86) and *in vitro* (87) experiments. The transformation of vascular smooth muscle cells into osteoblast-like cells is a key mechanism underlying vascular calcification in advanced atherosclerosis (88). Higher osteocalcin levels were observed in calcified plaques and aortic valves (89), and there was a positive correlation between vascular smooth muscle cell shift to osteoblast-like cells and osteocalcin expression (90). However, a clinical meta-analysis has indicated that in certain cardiovascular diseases, particularly atherosclerosis, no discernible correlation was observed between osteocalcin and markers of atherosclerosis and calcification (91). In contrast, studies of young adults have revealed a strong correlation between osteocalcin and cardiovascular disease, with this association becoming more pronounced with age (92). Given the role of osteocalcin in improving blood glucose, blood lipids and alleviating diabetes, its effects may be indirect and related to the regulation of blood glucose and lipid levels and metabolism. Despite the fact that numerous experiments have demonstrated that the role of osteocalcin in cardiovascular diseases is complex and still unclear, further research is required, the close association between osteocalcin and some diseases, in particular atherosclerosis, may prove to be a significant breakthrough in this field.

Given the regulatory role of osteocalcin in bone formation and remodeling, there has been considerable interest in its association with tumors, especially primary bone tumors. A reduction in osteocalcin expression has been observed in tumor cells in numerous animal experiments on osteosarcoma (93, 94). This may be associated with defective collagen fiber mineralization in osteosarcoma (95). Furthermore, rearrangements and loss of expression of the p53 gene have been observed in the vast majority of osteosarcomas (96). It has been postulated in animal studies that p53 rearrangement may contribute to the maintenance of a tumourigenic phenotype in osteosarcoma (93). Furthermore, it has been suggested that the p53 gene is a positive regulator of osteocalcin (97). It is therefore challenging to ascertain whether reduced osteocalcin levels contribute to elevated osteosarcoma incidence, whether increased osteosarcoma incidence is attributable to p53 rearrangements, or if both factors are involved. Further study is required to elucidate the precise effects of osteocalcin on osteosarcoma. While the precise role of osteocalcin in osteoblast differentiation remains unclear, its potential as a discriminating criterion for the degree of differentiation has been clinically validated (98). It is noteworthy that a viral toxicity therapy based on the intravenous injection of the osteocalcin promoter was observed to significantly inhibit the growth of osteosarcoma lung metastases and significantly improve survival in animal experiments (99). This appears to indicate that osteocalcin may also exert an inhibitory effect on osteosarcoma metastases. This offers a novel perspective on the potential treatment of osteosarcoma and its metastases.

### Differential expression of osteocalcin gene in relation to diabetes mellitus

Cluster 2 is characterised by the core terms ‘expression’, ‘differential’, ‘diabetes’ and ‘osteoblasts’. In this group, differential gene protein expression in osteoblasts may be associated with diabetes and the development of diabetic bone disease.

On the one hand, bone trap cells, osteoblasts produce rankl which is an important factor for osteoclast differentiation and activation (100), and osteoclasts have an extremely important role for the conversion of osteocalcin to ucOC, and ucOC has an effect on insulin that enhances its sensitivity phenotype, so that osteoblasts have an indirect regulatory role for insulin and glucose. In diabetic patients, high blood glucose decreases osteoblast and osteoclast activity, and Wittrant, Y. et al. showed that high blood glucose inhibits osteoclast formation, which may be due to the inhibition of redox-sensitive NF-kappaB activity through antioxidant mechanisms to alter RANKL-induced osteoclast formation (101).

Also, in osteoblasts, sympathetic tone stimulates the expression of Esp, a gene that inhibits osteocalcin activity by acting on its own to reduce osteocalcin secretion (102). In addition, the transcription factors forkhead box O1 (Foxo1) and activating transcription factor 4 (ATF4) inhibit osteocalcin metabolic activity by increasing ESP expression in osteoblasts (103). In a cross-sectional Danish study, Starup-Linde, J. et al. found that elevated non-fasting blood glucose levels were negatively correlated with p-CTX, p-P1NP, p-OC and p-ucOC, with the strongest effect being seen for hypo-carboxylated OC, which was reduced by 38% (104). In addition, the diabetes treatment drug, metformin, may ameliorate the inhibitory effect of hyperglycaemia on osteoblast proliferation and gene expression (105).

The role of inflammation in T2DM is unclear and may be indirectly mediated, whereby adipocytes can activate inflammation through the production of (ROS), which in turn increases the production of inflammatory cytokines that can reduce the number of osteoblasts and stimulate osteoclast bone resorption via apoptosis at the same time. Interestingly, this process becomes sustained as ROS stimulate MSCs to differentiate preferentially into adipocytes rather than osteoblasts, leading to a reduction in the transcription of Wnt proteins and further inhibiting bone formation (106).

### The role of osteocalcin in the evaluation of osteoporosis and diabetes

Cluster 3 is characterized by the core terms “osteoporosis”, “bone turnover”, “male” and “female”. This group primarily elucidates studies on the association between bone alkaline phosphatase levels and 42 mixed gender patients(male and femal), including research on osteoporosis and diabetes.

Firstly, osteoporosis is an imbalance between the activities of osteoclasts and osteoblasts. Factors influencing this imbalance include calcium intake (25), vitamin D receptors (107), vitamin D receptors (107), physical activity or mechanical stress (108), hormones (parathyroid hormone, testosterone, estrogen) (109), and the Receptor Activator Of Nuclear Factor-Kappa-B Ligand(RANKL) (110).

Secondly, osteocalcin, as a Bone Turnover Marker(BTM), may serve as one of the criteria for evaluating the risk of osteoporosis. In cross-sectional studies of women, an increase in osteocalcin(OC) may reflect enhanced bone turnover, partially contributing to the development of osteoporosis (111). Moreover, in the diagnosis of osteoporosis, bone turnover markers(btms) can be widely used to improve prognosis and monitor the response to anti-resorptive therapy (112). Therefore, serum osteocalcin may play a role in investigating patients with osteoporosis (113). Studies on men reveal that after the age of 60, an increase in bone resorption markers(rather than bone formation markers) is observed in some men, correlating with lower bone mineral density(BMD), indicating that this imbalance is a cause of increased bone loss in elderly men (114, 115). Retrospective studies show that low bone density in male osteoporosis is primarily due to reduced bone formation (116).

Ultimately, bone gla protein levels may serve as a potentially useful indicator for predicting the risk of type 2 diabetes(T2D) onset. In a controlled study of gestational diabetes in women, serum bone gla protein levels were elevated throughout pregnancy in women with gestational diabetes (117). In a cross-sectional analysis of postmenopausal women, bone gla protein levels were significantly lower in those with type 2 diabetes (118), and bone gla protein levels were significantly associated with the development of type 2 diabetes (79), suggesting that bone gla protein may play a role in regulating blood glucose levels in postmenopausal women (118). A cross-sectional study of men with type 2 diabetes found that bone gla protein was significantly correlated with glucose metabolism (119). Retrospective studies showed that in men with T2D, bone gla protein was negatively correlated with the triglyceride-glucose(tyg) index, a surrogate marker for insulin resistance (120), and undercarboxylated bone gla protein(ucoc) was associated with plasma glucose levels in men with type 2 diabetes (41), indicating that undercarboxylated bone gla protein may play a significant role in the pathogenesis of diabetes (119). Overall, bone gla protein levels may be a potentially useful indicator for predicting the risk of Type 2 Diabetes(T2D) onset (121, 122), but further research is needed.

It is worthy of note that a particular form of osteoporosis, known as diabetic osteoporosis, merits specific mention. Disturbed calcium and phosphorus metabolism in diabetic patients (123) results in abnormal collagen cross-linking, which in turn leads to reduced bone strength and increased bone fragility (124). Patients with diabetic osteoporosis are at a higher risk of sustaining a fracture than patients with normal osteoporosis (125), and the healing time for such fractures is significantly longer (126). The prevailing view on diabetic osteoporosis is currently based on the oxidative stress theory (127), which posits that excess reactive oxygen species (ROS) disrupt antioxidant defense mechanisms, leading to increased bone loss and inhibition of bone formation and inducing apoptosis in osteoblasts (128). This inflammatory mechanism is dependent on NOX2 (129). Given its involvement in bone remodeling and maturation, low osteocalcin levels are associated with reduced bone resorption, increased bone fragility, and a markedly elevated risk of fracture (130). Despite the long-standing use of total osteocalcin as a bone turnover marker for the assessment of bone turnover status (131), however, it has also been proposed that the accuracy of this method may be questionable, with no significant difference in total osteocalcin levels observed between osteoporotic and healthy individuals (132). Nevertheless, further investigation is warranted to elucidate the potential association between osteocalcin and diabetic osteoporosis. Furthermore, although the correlation between low osteocalcin levels and the onset of diabetes is not consistent across studies (133, 134), it would be beneficial to investigate this link in larger groups. This could provide insights into the specific mechanisms of diabetic osteoporosis.

### Osteocalcin’s indirect involvement in metabolic processes

Finally, “Osteocalcin action on islet cells and adipocytes” is the topic of Cluster 4, which includes the keywords “osteocalcin”, “metabolism”, “insulin resistance” and “plasma glucose”. For example, in the study investigating the association between osteocalcin and time interval (TIR), researchers focused on the correlation between osteocalcin and continuous glucose monitoring (CGM) metrics in patients with type 2 diabetes mellitus (T2DM) (135). This topic focuses on the potential of osteocalcin as a new direction in the treatment of diabetes.

To gain a full understanding of this topic, we will look at two types of studies: animal and clinical.

Osteocalcin was shown to regulate insulin in animal studies as early as 2007 (48), and a large number of studies have now elucidated the associated regulatory signalling mechanisms and functional roles, particularly in mouse models. Currently, much of the research in this area is focused on changes in osteocalcin levels in diabetic animal models in response to interventions aimed at promoting bone regeneration and thus better treating osteoporosis, one of the complications of diabetes. In a diabetic rat model of critical size defects in the skull, osteocalcin expression levels can be increased by the administration of vitamin K2 , which facilitates bone healing (136). Similarly, many drugs may have potential clinical therapeutic value, such as mogroside V (MV), which promotes the ability of bone marrow mesenchymal stem cells (BMSCs) to differentiate into osteoblasts in diabetic mice (137), Icariin (ICA), which induces osteogenic-angiogenic coupling in a rat model of type 1 diabetic bone defects (138), and the combination of insulin (I) and naringin (NAR), which prevented osteoclast proliferation in type 1 diabetic rats (139). In insulin-targeted tissues, ucOC can increase glucose and fatty acid uptake (10), insulin sensitivity (11), nutrient utilisation and mitochondrial capacity, and reduces glycogen production in muscle and lipid synthesis in the liver (12, 13). Moreover, non-carboxylated osteocalcin ucOC is regulated by insulin and increases β-cell proliferation as well as insulin production and secretion, whereas skeletal muscle and adipose tissue respond to osteocalcin by increasing insulin sensitivity (14). In the pancreas, ucOC has been demonstrated to directly promote β-cell proliferation and insulin secretion via GPRC6A (7, 8). Additionally, ucOC has been shown to indirectly promote insulin secretion by increasing intestinal glucagon-like peptide-1 (GLP-1) levels, which in turn promotes GPRC6A secretion (9). And insulin also has the capacity to elevate ucOC levels via insulin receptors in two distinct ways. Firstly, insulin inhibits the expression of the Runx2 repressor Twist2, which in turn stimulates bone formation and osteocalcin production (140). Secondly, insulin reduces the production of the anti-osteoclastic factor osteoprotegerin. This results in an increase in the number of osteoclasts and enhanced bone resorption. The subsequent acid environment of the bone resorption traps (pH 4.5) promotes bone matrix decarboxylation of osteocalcin (27). This results in a positive feedback regulatory pathway between insulin and osteocalcin. UcOC also enhances the production of delta like-1 (DLK1) in pancreatic β-cells, which inhibits insulin-dependent OC production in osteoblasts (141), which in turn negatively feedback regulates ucOC production, thereby maintaining insulin levels within a reasonable range.

Many clinical studies also support the existence of the same complex regulatory mechanism between non-carboxylated osteocalcin and insulin in humans. In elderly subjects, negative correlations between serum ferritin and soluble transferrin receptor (sTfR) and osteocalcin led to the conclusion that factors related to iron metabolism may contribute to the development of insulin resistance and type 2 diabetes (142). In obese middle-aged men, insulin sensitivity after acute exercise is associated with individual levels of circulating ucOC (143). Changes in sub-glucocorticoid-induced osteocalcin concentrations were associated with reduced hepatic insulin sensitivity in a randomised controlled trial in normal men (144). In young adult Japanese patients with childhood-onset type 1 diabetes mellitus, serum osteocalcin concentrations were negatively correlated with patient body fat. By recruiting subjects from the Shanghai community, it was also concluded that serum osteocalcin levels were significantly lower in patients with diabetes mellitus, overweight/obesity or central obesity than in patients without diabetes mellitus (all P<0.01) (145).

Similarly, some studies were unable to establish a causal relationship between changes in osteocalcin and incident diabetes and insulin levels. There were no clinically important effects of antiresorptive therapy to reduce ucOC levels on fasting glucose, body weight or risk of diabetes in postmenopausal women (146). Another study also showed that changes in body composition measures or markers of fat or glucose metabolism in postmenopausal women were not associated with changes in non-carboxylated osteocalcin. This suggests that the effect of osteocalcin may be influenced by a variety of factors such as the metabolic state of the subject, other endocrine regulation and other factors (147).

Future studies need to further explore the fine regulation mechanism of osteocalcin in the human body and how it can be effectively used as a target for the treatment of metabolic diseases. In particular, the therapeutic potential of osteocalcin for diabetes and other metabolic diseases should be validated by large-scale, multicentre, randomised controlled trials.

### Hot topics

Current research focuses on three main areas: the association between osteocalcin levels and diabetic complications, the clinical efficacy of drugs or vitamins for osteoporosis in diabetic patients of different ages and sexes, and the potential mechanism of insulin regulation by osteocalcin. Recent studies have correlated it with diabetic complications such as atherosclerosis (147) and cataracts (148) to provide a more accurate guide for prognostic assessment and therapeutic strategies in diabetic patients. Therefore, recent studies have focused on evaluating the clinical efficacy of different drugs or vitamins for osteoporosis in diabetic patients in order to explore the optimal therapeutic regimen and optimise patients’ bone health. In addition, as osteocalcin is thought to influence the pathogenesis and progression of diabetes mellitus by regulating insulin secretion and sensitivity, future studies may aim to further explore the role of osteocalcin in the development of diabetes mellitus. Future studies may be devoted to exploring the fine regulatory mechanisms between osteocalcin and insulin metabolism, such as signalling pathways and gene regulatory mechanisms in the human body, to reveal its specific biological functions in the pathogenesis of diabetes and to provide theoretical basis and experimental evidence for the development of novel therapeutic strategies.

### Strengths and limitations

Inevitably, there are some limitations to this study. Firstly, relevant articles were screened only in the WOS database, which is the most widely used database in the world. Scopus and other databases such as SciELO, Google Scholar, and Scopus were not included in the search. Secondly, our study focused exclusively on original research and other types of publications were excluded, which may have led to omissions. Third, authors were analysed differently based on their bylines. However, over the years, some authors can have sign their papers in different ways and can create important biases in such analyses. Fourth, changes in author affiliation or dual affiliation at the time of publication may also create difficulties when analysing the data. In this study, we are cautious about these limitations. Finally, due to the low number of citations to some high-quality literature published in recent years and the fact that the databases are kept open, new papers published after the search date were not included in the study, which could have been lost in the vast ocean of literature data.

## Conclusion

**Figure 11:**
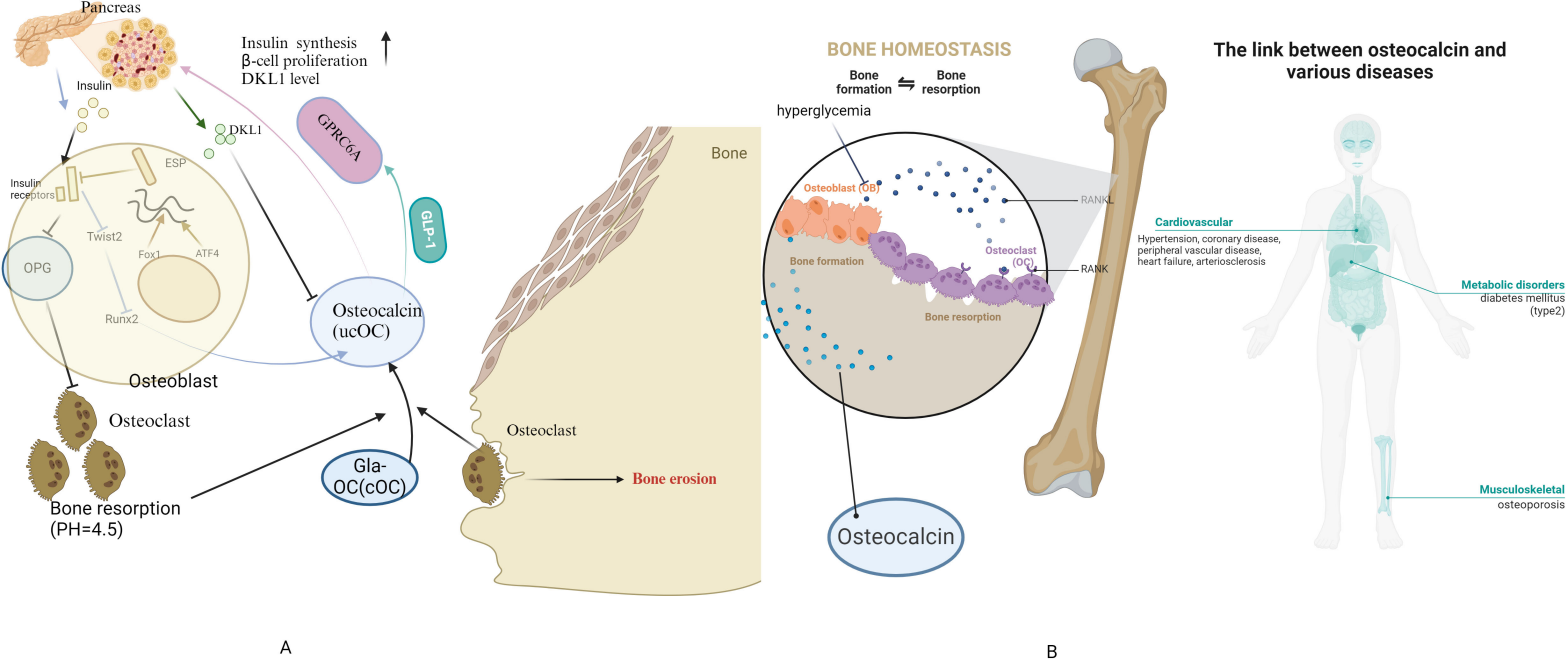
Summary of the osteocalcin **(A)** Physiological mechanisms of osteocalcin-insulin interactions **(B)** link between osteocalcin and diseases.

The number of publications in the field of diabetes and osteocalcin research has increased significantly since 2013. Mainland China and the United States have become high-productivity countries, while the countries with the highest article citations are the United States and Japan. As research has progressed, the focus has shifted to the relationship between osteocalcin levels and diabetic complications, the clinical efficacy of drugs or vitamins for osteoporosis in diabetic patients, and the potential mechanism of insulin regulation by osteocalcin(as shown in **Figure 11**-summary of the osteocalcin). To make substantial progress in this area, it is recommended that the terms “osteocalcin gene”, “osteoporosis”, “type 2 diabetes mellitus”, “different populations”, “older men”, “pregnant women”, “diabetes-related complications”, “treatment and correlation studies” and other key topics.

## Data Availability

The original contributions presented in the study are included in the article/supplementary material. Further inquiries can be directed to the corresponding author.
